# Clinical investigation protocol for tinnitus (CIP-t): development and validation of an instrument

**DOI:** 10.1590/2317-1782/e20250125en

**Published:** 2026-04-27

**Authors:** Hélinton Goulart Moreira, Michele Vargas Garcia

**Affiliations:** 1 Programa de Pós-graduação em Distúrbios da Comunicação Humana, Departamento de Fonoaudiologia, Universidade Federal de Santa Maria – UFSM - Santa Maria (RS), Brasil.

**Keywords:** Tinnitus, Protocol, Assessment, Adults, Older Adults

## Abstract

**Purpose:**

The objective of this study was the development and validation of a clinical investigation protocol for tinnitus and the analysis of its applicability.

**Methods:**

The research was conducted in eight stages: Stage 1: Literature review and development of the pilot protocol; Stage 2: Expert judges I – first analysis/evaluation; Stage 3: Elaboration of the unified investigation protocol and the application guide; Stage 4: Expert judges II – second analysis/evaluation of the instruments from the previous stage; Stage 5: Refinement of materials; Stage 6: Application of the protocol by expert and non-expert clinicians; Stage 7: Final construction; Stage 8: Pilot study – convenience sample. The results of each stage were compiled into an Excel spreadsheet for specific analyses (content validity ratio calculation and suggestions).

**Results:**

The structure of the protocol was successfully developed, resulting in a unified instrument subdivided into: tinnitus-focused anamnesis, audiological, emotional, metabolic, somatosensory, and vascular history, assessment results, a framework for subgroup classification, clinical decision tree, and complementary evaluations. The protocol was applied to a sample of 83 adults and older individuals.

**Conclusion:**

The clinical investigation protocol for tinnitus was finalized and its content validated, demonstrating applicability for adult and elderly individuals with tinnitus.

## INTRODUCTION

Chronic tinnitus is an otological symptom with multiple causes and an incidence of approximately 740 million adults worldwide. It is perceived as a major problem by more than 120 million people, mainly in individuals over the age of 65^(1)^.

Initially, its pathophysiology was explained by the neurophysiological model, highlighting its peripheral origin, detected at the level of the central auditory nervous system, causing discomfort when associated with the limbic system. Given its manifestations, a recent study suggested that it should be classified as a disorder when associated with cognitive impairment, speech perception impairment, and/or emotional changes^(2)^.

The multifactorial etiology that can trigger and influence it makes each subject unique. Thus, for the intervention process to be effective, a comprehensive assessment program and individualized treatment are necessary^(3-5)^. In this sense, public health managers should consider the global impact of tinnitus, investing in more research and public policies to boost its investigation^(1)^.

Given its high prevalence and the negative impact it has on individuals' lives, tinnitus can be considered a public health issue and needs to be included in planning at all levels of health care. Another relevant aspect is the lack of professional knowledge, as most professionals, even those specializing in audiology, do not consider themselves qualified for the assessment and clinical management of this population^(6)^.

The lack of knowledge among professionals points to the difficulties that patients are likely to encounter in identifying the most appropriate health care. A journey of uncertain treatments and a lack of clear referral pathways are likely to be factors that aggravate the suffering, severity, and chronicity of tinnitus. One possibility for addressing these gaps is the creation of protocols that focus on the initial care provided to patients by health professionals. These documents address topics such as prevention, health promotion, management of chronic conditions, and referrals to specialists, ensuring that care is integrated and coordinated effectively.

This study is justified by the lack of an integrative instrument in Portuguese for detailed investigation of tinnitus subgroups, seeking to measure the different etiological factors that generate and amplify the symptom, which are most often associated. These can be auditory, emotional, vascular, somatosensory, and/or metabolic, and need to be well defined in the clinical evaluation for better therapeutic assertiveness^(4)^. By treating the cause of the symptom, the possibility of remission or reduction of the disorder caused by tinnitus increases.

The hypothesis is that, through a detailed clinical evaluation, a more effective treatment can be achieved, due to the multifactorial etiology of tinnitus, that is, by identifying the agents of the symptom, it is possible to perform a more appropriate treatment^(5,7,8)^. Therefore, the objective of the study is to develop a protocol and clinical application guide for tinnitus, as well as to analyze its applicability.

## METHOD

### Ethical aspects

This is a qualitative-quantitative, cross-sectional study approved by the Research Ethics Committee of the Federal University of Santa Maria (UFSM), under number 57700721.0.0000.5346. It should be noted that the research complied with all guidelines for conducting research with human subjects in force in Resolution 466/12 of the Brazilian National Health Council. All participating judges and individuals in the pilot study signed a specific Free and Informed Consent Form, granting their participation in the research.

Participants in the protocol were informed about the risks and benefits of their participation. All assessments were performed at the Audiology Clinic of a higher education institution. In addition, individuals diagnosed with tinnitus disorder were welcomed and received treatment at the institution's specialized tinnitus clinic.

### Procedures and participants

To ensure a better methodological understanding, the research was structured in phases and stages. Chart 1 presents a timeline of the phases, stages, participants, and selection criteria.

**Chart 1:** Description of participants and selection criteria for each stage of the process of creating and validating content for the Clinical Investigation Protocol for Tinnitus (CIP-t)

CONTENT CONSTRUCTION AND VALIDATION			
Phases	Stages	Participant	Selection criteria
Phase 1	Stage 1: Literature review and development of the pilot protocol	02 speech therapists	Study authors.
	Step 2: Expert judges I - first analysis/evaluation of the pilot protocol	03 speech therapists 01 physical therapist 01 physician	Doctors with at least 10 years of clinical and research experience, all in the field of tinnitus.
	Stage 3: Development of a single research protocol and application guide.	02 speech therapists	Authors of the study.
Phase 2	Stage 4: Expert judges II - second analysis/judgment of the protocol and application guide (same as in stage 2)	03 speech therapists 01 physical therapist 01 physician	Doctors with at least 10 years of clinical experience, all in the field of tinnitus.
	Stage 5: Adjustments to the protocol and application guide.	02 speech therapists	Authors of the study.
	Stage 6: Specialist and non-specialist judges - clinical application	Specialists: 04 speech therapists 01 physical therapist Non-specialists: 04 speech therapy students 01 medical student	Professionals with at least 2 years of clinical experience in audiology or tinnitus. Students with less than one year of experience with tinnitus.
	Stage 7: Final construction of the CIP-t	02 speech therapists	Authors of the study.
Phase 3	Stage 8: Pilot study - convenience sample	83 individuals	Adults and elderly individuals, aged between 17 and 80 years, with perception of tinnitus.

**Caption:** CIP-t = Clinical Investigation Protocol for tinnitus

The methodological steps are described in detail below, according to psychometric principles related to content construction and validation.

### Phase 1 – Protocol development and preparation of an application guide

In this phase, a protocol was developed for health professionals, integrating various tinnitus investigation instruments. This phase comprised steps 1, 2, and 3 of the content construction and validation process.

In step 1, a literature review was conducted by the authors of the research to develop a pilot protocol with the suggested assessments. This protocol, together with an assessment questionnaire, both developed by the authors, were sent to five expert judges (three speech therapists, one physical therapist, and one physician), all of whom were doctors with at least ten years of clinical and research experience in the field of tinnitus, selected to perform the initial assessment.

The judges were chosen based on their experience in the area of instrument development, as well as their prior knowledge of the methodological processes involved in the construction of new tools^(9)^.

Stage 2 consisted of the first analysis performed by the expert judges. After sending their responses to the study authors, the considerations were tabulated in an *Excel* spreadsheet and the Content Validity Ratio (CVR) was subsequently calculated. The CVR analysis, as proposed by Lawshe^(9)^, considers the degree of agreement among the evaluators regarding the relevance of each item in the instrument. The items are categorized into three levels: essential, useful but not essential, and non-essential. To calculate the CVR, the number of judges who classified the item as essential (ne) and the total number of evaluators involved in the analysis (N) were used, applying the following formula: CVR = (ne – N/2) / (N/2). It should be noted that the CVR calculation was considered adequate for all stages when it obtained values of ≥ 0.99, according to the number of participating judges^(9)^.

Based on these analyses, the study authors created the first version of the protocol in a single format and the application guide, constituting stage 3.

### Phase 2 – Validation of the protocol by experts

The protocol was validated through analysis by expert judges, non-expert judges, and authors. This phase covered steps 4, 5, 6, and 7 of the methodological process.

Step 4 consisted of a second analysis of the protocol and application guide, carried out by the same expert judges as in step 2. Step 5 was carried out by the study authors, who measured a new CVR calculation and analyzed the judges' suggestions, which were used for initial adjustments to both documents (protocol and application guide).

After the adjustments, stage 6 began with the clinical application of the structured protocol and analysis of the application guide. This stage was carried out by five expert judges (four speech therapists and one physical therapist, with at least two years of expertise in tinnitus) and five non-experts (four speech therapy students and one medical student, with more than three months and less than one year of experience with individuals with tinnitus), aiming to measure the clinical applicability of the instrument.

Finally, stage 7 was carried out by the authors of the study, through adjustments and final construction of the Clinical Investigation Protocol for Tinnitus (CIP-t) and the Application Guide.

### Phase 3 – Applicability of the protocol in a sample

In this stage, corresponding to stage 8, the protocol was validated with a convenience sample, using the cross-sectional observational method. Thus, individuals who sought care at the institution's teaching clinic participated in the study, with perceptions of unilateral or bilateral tinnitus of different durations (chronic or not), perceptions, locations, and manifestations, with the objective of analyzing the applicability of the instrument in a real data collection situation.

The CIP-t was applied by the study authors and members of the Research Group at the Speech Therapy Service-UFSM, after training. The instrument was administered in a single room with an acoustically treated booth and sufficient physical space. The audiological diagnosis was performed using the following equipment: *Mikatos* TK otoscope, supra-aural headphones (*Telephonics* TDH-39), AD629B audiometer, and AT235 immittance meter, both from *Interacoustics*. In the same environment, the assessments and questionnaires were administered. For the somatic and vascular assessments, the individuals were seated in a comfortable, reclining chair. After administration, the data were entered into an *Excel* spreadsheet for subsequent analysis of the protocol findings.

The CIP-t was applied to a pilot sample of 83 adults and elderly individuals of both sexes (55 women and 28 men), aged 17 to 80 years (mean age = 51.77 years; standard deviation = 26.35) and with perception time ranging from one to 600 months (mean months = 91.42; standard deviation = 105.64). Of these, 73 had constant manifestations and 10 had intermittent manifestations. Furthermore, among the individuals evaluated, one reported acute perception (less than three months), 74 reported chronicity, and eight were unable to say when the symptom first appeared.

Table 1 shows the manifestations and characteristics related to tinnitus.

**Table 1:** Sample characteristics related to tinnitus (N=83)

Category	Description	N (total subjects)
Location	Head	9
	Right ear	7
	Left ear	12
	Bilateral	11
	Both, worse on the right	30
	Both, worse on the left	14
Type of perception	Single	71
	Multiple	12
Single perceptions (n=71)	Whistle	23
	Cicada/Cricket**	12 (each)
	Bee	6
	Hissing / Pressure cooker**	4 (each)
	Waterfall	2
	Ambulance / bee wing / heart / motor / breath / tractor / jet turbine / windstorm**	1 (each)
Multiple perceptions (n=12)	Cicada + whistle	4
	Whistle + cricket	3
	Cricket + Cicada	2
	Whistle + pressure cooker	1
	Waterfall + Bee	1
	Waterfall + Cricket	1
Tonal frequency of the buzzing sound	High	57
	Medium	16
	Low	10

** They were grouped together because they had the same incidence (example = cicada/cricket obtained 12 each, totaling 24 subjects)

Table 2 shows the discomfort, volume, and degree of tinnitus among the participants, which were moderate.

**Table 2:** Description of discomfort, volume, and degree of tinnitus among participants (N=83)

Measure	Mean	Minimum	Maximum	Standard deviation
VAS- Discomfort	6.84	2	10	3.56
VAS- Loudness	6.49	2	10	3.41
THI- total	46.77	10	100	21.73
THI- functional	19.90	4	44	12.59
THI - emotional	17.24	0	38	11.22
THI- catastrophic	10.80	0	40	7.48

**Caption:** VAS = visual analog scale; THI = *tinnitus handicap inventory*

It is noteworthy that all participants were able to complete the assessments without showing fatigue, only discomfort when performing finger pressure. The protocol was applied and completed with an average time of approximately one hour and thirty minutes. Therefore, the items in the instrument were sufficient to meet the objective of the assessment.

## RESULTS

### Stage 1 – Literature review and development of the pilot protocol

In this stage, three studies were used: Tunkel et al.^(3)^, Onishi et al.^(4)^ and Cima et al.^(8)^, selected due to their comprehensiveness in the assessments and scientific relevance. The chosen assessments are described below.

- Clinical history: Used for the initial diagnosis and intervention process. Thus, the medical history of tinnitus investigation was selected.
- Questionnaires and scales: Necessary for understanding and distinguishing the perception of tinnitus from tinnitus disorder. Thus, the *Tinnitus Handicap Inventory* (THI) - repercussion of tinnitus on quality of life (degree, functional, emotional, and catastrophic impacts) and the Visual Analog Scale (VAS) - intensity and volume of the symptom were selected.
- Audiological evaluation: Used in the diagnosis of hearing changes, guidance, and intervention. Visual inspection of the external auditory canal, pure tone audiometry (250 to 8000Hz), speech audiometry (speech recognition threshold and speech recognition percentage index), and acoustic impedance measurements (immittance testing and contralateral stapedial acoustic reflexes) were selected.

The following assessments are recommended to investigate predisposing or contributing factors in the perception of tinnitus. It is important to note that, although emotional screening, vascular and somatosensory assessments, or requests for laboratory tests may be performed by speech therapists in order to establish subgroups related to perception, they will not be conducted by such professionals (CFFa No. 246/2000)^(10)^. The main objective is to identify amplifying factors, enabling accurate referrals.

- Somatosensory assessment: This should be performed by a qualified and trained professional, due to the time and force required to apply each technique. It is used to investigate somatic influences on the perception of tinnitus related to the head, face, and neck regions. The following assessments were selected as possible options when associated with the four diagnostic criteria proposed by Michiels^(11)^: digit-pressure/palpation modulation, intra-duct maneuver, mandibular movements, cervical movements, and ocular movements^(11)^.
- Emotional screening: This can be applied by any health professional and investigates emotional factors, mainly anxiety and depression as amplifiers and aggravators of tinnitus. To achieve this objective, the Hospital Anxiety and Depression Scale (HADS) was selected^(12)^.
- Laboratory tests: The following tests were selected: complete blood count, fasting blood glucose, glycated hemoglobin, total cholesterol and fractions, triglycerides, Glucose, Insulin, Free T4, TSH, Zinc, Magnesium, Vitamin B12, Vitamin B9, Cortisol, Serotonin, Vitamin D, Vitamin A, Vitamin C, Iron, Ferritin, Calcium, which may be etiological, predisposing, or contributing factors^(4)^.
- Vascular screening: Used to correlate possible vascular and cardiac changes with pulsatile tinnitus, such as malformations, arteriovenous fistulas, intracranial hypertension, and flow changes, which can cause pulsatile tinnitus. Thus, carotid artery compression and head lateralization to both sides were selected^(13)^. The diagnosis of vascular changes is made by the physician, and screening only serves to guide the patient.
- Highly recommended audiological evaluations: these were suggested to investigate etiological factors not evident in the basic audiological evaluation. The objective is to measure the individual's demands or analyze self-reported complaints. Thus, the following were selected:
- Psychoacoustic evaluations of tinnitus (tinnitus measurement—frequency and intensity, minimum masking level, and residual inhibition). These measurements can be used to inform the patient about their symptoms, in counseling, in monitoring treatments, and in recommending treatments related to sound therapy.
- High-frequency audiometry - 9000 to 20,000Hz and/or Transient Otoacoustic Emissions and/or Distortion Product Otoacoustic Emissions, subject to normal results in conventional audiological assessment.
- Auditory discomfort threshold - 250 to 8000Hz with pure tone and speech for those subjects complaining of discomfort with sounds.
- Eustachian tube function test and self-perception questionnaire for individuals complaining of ear fullness.
- Brainstem auditory evoked potentials and long latency for differential diagnosis and understanding of the neurophysiological changes resulting from the symptom.

In light of national and international recommendations, the pilot protocol was developed based on the review. A *Word* document was compiled with the protocols and assessments, along with guidelines for implementation, and sent to judges for analysis.

### Stages 2 and 3 – Specialist judges I (first analysis) and development of the Single Investigation Protocol and Application Guide

In general, in stage 2, changes were necessary in all the assessments suggested in the pilot protocol and sent to the expert judges.

In the anamnesis, an CVR of 0.2 was evident, with changes suggested regarding the insertion of personal data, separation of the protocol into research topics, and inclusion of questions about tinnitus, sound perception disorders, and tensor tympani syndrome.

In the questionnaires and scales, an CVR of 0.6 was observed, with only the inclusion of a questionnaire that assesses the quality of life in subjects with tinnitus being suggested, as it presents more domains.

Regarding the audiological assessment, it was recommended to include the assessment of auditory and cognitive skills, discomfort threshold, residual inhibition, and the creation of a space for previous audiological results, demonstrating an CVR of 0.4.

For emotional screening, the inclusion of two more questionnaires as possible applications was suggested (CVR = 0.8).

In the somatosensory assessment, an CVR of 0.6 was observed, due to the need to include assessments in the physical examination, add muscle regions, remove the intracanal maneuver, and the suggestion that the somatosensory assessment be performed only by a physical therapist.

Finally, for both metabolic and vascular assessments, an CVR of 0.4 was observed, for which the judges suggested the inclusion of requests for new laboratory tests and complementary imaging exams, and one judge recommended that these be requested only by medical professionals.

In questions about the applicability of the protocol, focused on the measurement of subgroups, an CVR calculation of 0.6 was obtained, due to the suggestion that the somatosensory assessment be performed by the physical therapist and the vascular and metabolic assessments by the medical professional. However, in professional management, it was 0.8, due to the assessment of tinnitus disorder being performed in a multidisciplinary manner. Regarding the clinical outcome for the application of the protocol for inclusion in clinical practice, an CVR with 100% agreement was obtained, with no suggestions (auditory and emotional subgroups). Finally, given the majority of judges who accepted these screenings, even though they did not reach the expected CVR for vascular, metabolic, and somatosensory, they were maintained in the protocol.

In stage 3, the instrument was developed in a single format. It was subdivided into: identification and general information, part I: investigation history, part II: evaluation results, and part III: etiological subgroups and decision tree. It was structured in this way so that the evaluator could access and record the patient's personal information, as well as the questions to be asked during the investigation, space to note the complaints for each subgroup, and a description of the assessments to be performed on all individuals and those highly recommended based on the complaints presented.

In part III of the protocol, a table of the etiological agents of perception was created, with the generators and amplifiers of the symptom. The objective was for this to contribute to the clinical evaluation, that is, for the evaluator to be able to understand the subgroups and whether there is a need for a specialized evaluation. Thus, the option “probable” refers to the certainty of the influence of a given subgroup, “unlikely” refers to the need for an evaluation by a specific professional, and “improbable” refers to the certainty that there is no influence. In addition, a decision tree was developed, which, through evaluation, guides the evaluator to the most appropriate professional for clinical management.

Given the complexity of the assessments and the suggestions of the expert judges, it was decided to create an Application Guide. This tool was developed to assist in completing and applying the protocol and interpreting the assessments, as it contains specific guidelines, the necessary tools, and the correlation of clinical findings.

### Stages 4 and 5 – Expert judges II (second analysis) and adjustments to the CIP-t and Application Guide

At this stage, no suggestions for changes to the protocol were made, demonstrating a 100% CVR index among the judges. On the issue of management and referral, an CVR of 0.8 was evident, as one judge stated that application and clinical management would depend on the clinician's expertise. Therefore, no adjustments were made to the protocol.

### Stage 6 – Specialist and non-specialist judges - clinical application and Stage 7 – Final adjustments to the application protocol

In this application stage, specialist and non-specialist judges suggested specific changes to the protocol, which are described and configured in stage 6.

Regarding the expert judges, for clarity of items, ease of application, insertion into clinical practice, diagnosis of subgroups, and referrals, a CRC of 100% was obtained, with no suggestions.

For the application time, it was 0.6, suggesting that emotional and somatosensory screening should only be performed on subjects with clinical signs or that a summarized version should be developed. For professional application, it was 0.2, due to the need for a speech therapist to always be on the team (audiological evaluation), the performance of specific evaluations for a given professional, and the need for clinical expertise for application and interpretation. Similarly, for the question about training prior to application, an CVR of 0.8 was obtained.

In the space provided for suggestions, the judges recommended specific additions, such as investigating in greater detail the type of exercise practiced, whether tinnitus changes with physical exertion, fasting time, and inclusion of the option without modulation in the somatosensory evaluation.

Regarding non-specialist judges, there were no suggestions regarding the clarity of the items, insertion into clinical practice, prior training for application, diagnosis of subgroups, and referrals, with an CVR of 100% agreement. The application time was 0.6, suggesting that the muscle assessment points be reduced or that it be applied in two sessions. The ease of application score was 0.8, emphasizing that, to facilitate the process, the points in the somatosensory assessment should be inserted in image format. In the multiprofessional application, the CVR was 0.4, due to the need for clinical expertise and the speech therapist always being part of the team. For the space for suggestions, it was suggested to include the medications used and the qualitative change in perception in the somatosensory assessment.

**Figure 1:**
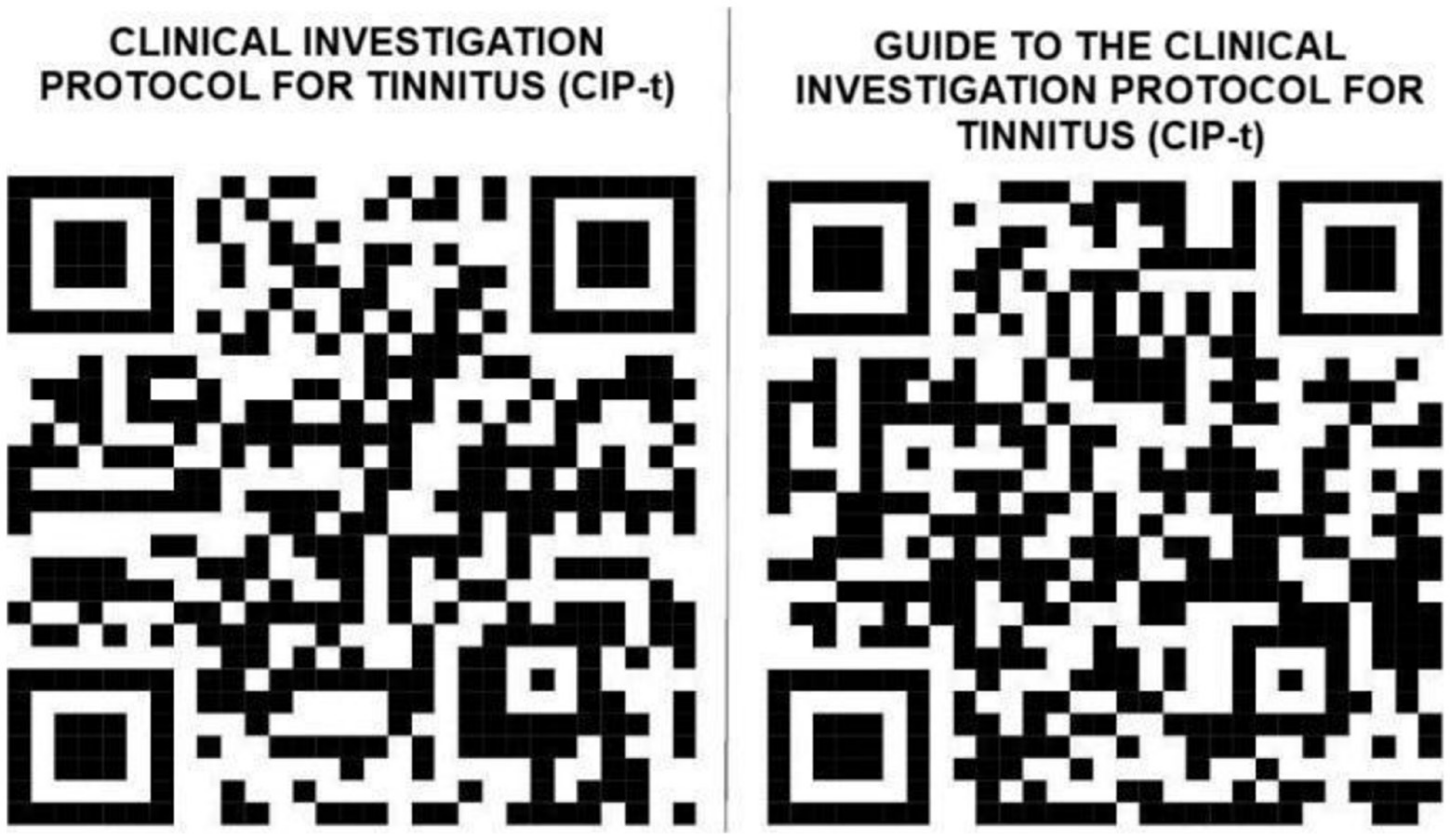
QR Code for access to the Clinical investigation protocol for tinnitus (CIP-t) and Application Guide

After analysis and changes made by the study authors, this led to stage 7, which consists of finalizing the version of the content, appearance, and application guide available in Figure 1.

### Stage 8 – Pilot study

Regarding the findings of the protocol application, Table 3 shows the incidence of etiological agents, with a higher percentage for the auditory, emotional, somatosensory, and metabolic subgroups.

**Table 3:** Characterization of participants considering the total sample for the different subgroups

**SUBGROUPS**				
	**Total number of subjects**	**Thresholds up to 8kHz**	**N**	**%**
HEARING	83	Normal	13	15.66
		Impaired	70	84.34
	**Total number of subjects**	**Emotional screening**	**N**	**%**
EMOTIONAL	83	Normal	12	14.46
		Altered	71	85.54
	**Total number of subjects**	**Laboratory tests**	**N**	**%**
METABOLIC	13	Normal	1	7.70
		Altered	12	92.30
	**Total number of subjects**	**Muscular influence**	**N**	**%**
SOMATOSENSORY	83	No	50	60.24
		Yes	33	39.76
	**Total number of subjects**	**Vascular influence**	**N**	**%**
VASCULAR	83	No	83	100
		Yes	0	0

**Caption:** kHz = thousand hertz; N = number of participants; % = percentage

Table 4 shows the findings of the audiological evaluation, with a higher incidence of hearing loss in both ears, of the sensorineural type and mild degree or in high frequencies (from 3KHz).

**Table 4:** Findings from the hearing subgroup assessment

HEARING SUBGROUP				
	**Total number of subjects**	**Ears**	**N**	**%**
Location	70	RE	3	4.29
		LE	2	2.86%
		BE	65	92.85%
	**Total number of ears**	**Type**	**N**	**%**
Right ear	68	Sensorineural	37	54.41
		Conductive	0	0
		Mixed	5	7.35
		Frequencies	28	38.24
Left ear	67	Sensorineural	39	58.21
		Conductive	2	2.99
		Mixed	5	7.46
		Frequencies	21	31.34%
	**Total number of ears**	**Grade** *	**N**	**%**
Right ear	68	Mild	27	67.50
		Moderate	5	12.50
		Moderately severe	4	10
		Severe	1	2.50
		Profound	2	5.00
		Complete/deaf	1	2.50
		**Total number of changes**	**40**	**100**
	**Total number of ears**	Grade*	**N**	**%**
Left ear	67	Mild	29	67.44
		Moderate	7	16.28
		Moderately severe	3	6.98
		Severe	4	9.30
		Profound	0	0
		Complete/deaf	0	0
		**Total number of changes**	**43**	**100**
	**Total number of ears**	**Frequencies (Hz)**	**N**	**%**
Right ear	68	250	9	10.34
		500	7	8.04%
		1000	0	0
		2000	9	10.34
		3000	11	12.64
		4000	17	19.55
		6000	18	20.67
		8000	16	18.39
		**Total number of changes**	**87**	**100**
	**Total number of ears**	**Frequencies (Hz)**	**N**	**%**
Left ear	67	250	4	5.00
		500	3	3.75%
		1000	0	0
		2000	3	3.75
		3000	14	17.50
		4000	19	23.75
		6000	19	23.75%
		8000	18	22.50
		**Total number of changes**	**80**	**100**

* For this analysis, subjects were classified according to the quadri-tonal average (500, 1000, 2000, and 4000Hz)

**Caption:** RD = right ear; LE = left ear; BE = both ears; Hz = Hertz; N = total number; % = percentage

Regarding the emotional complaints described in Table 5, the presence of anxiety and depression is evident, followed by stress.

**Table 5:** Self-reported complaints for the emotional subgroup

**EMOTIONAL SUBGROUP**				
	**Total number of subjects**	**Complaints**	**N**	**%**
Medical history	71	No complaints	17	16.50
		Anxiety	44	42.72
		Stress	14	13.59
		Depression	19	18.45
		Panic/fear/phobia	8	7.77
		Other	1	0.97
		**Total number of complaints**	**103**	**100**

**Caption:** N = total number; % = percentage

Table 6 shows the emotional assessment, demonstrating the presence of anxiety and depression, with a moderate score.

**Table 6:** Findings from the emotional assessment

Hospital Anxiety and Depression Scale (HADS)				
Analyses	Total N	Mean	Minimum-Maximum	Standard deviation
Total	71	15.28	1 -30	9.35
Anxiety		8.42	0 - 16	5.0
Depression		6.86	0 - 17	4.87

**Caption:** N = total number

Table 7 shows the laboratory measurement findings, with altered rates for blood count, cholesterol, and vitamin D.

**Table 7:** Metabolic assessment findings

METABOLIC SUBGROUP				
	Total number of subjects	Laboratory tests	N	%
Laboratory rates	12	Complete blood count	3	12
		Blood sugar	2	8
		Cholesterol	5	20
		Triglycerides	2	8
		Glucose	2	8
		TSH	2	8
		Cortisol	1	4
		Iron	1	4
		Ferritin	1	4
		Vitamin C	1	4
		Vitamin D	4	16
		Zinc	1	4%
		**Total Fees**	**25**	**100**

**Caption:** N = total number; % = percentage

Regarding the self-reported musculoskeletal complaints shown in Table 8, there was a higher incidence of pain and temporomandibular dysfunction.

**Table 8:** Self-reported musculoskeletal complaints in the somatosensory subgroup

**SOMATOSENSORY SUBGROUP**				
	**Total number of subjects**	**Complaints**	**N**	**%**
Medical history	**33**	Pain	23	40.35
		Blood pressure	8	14.04
		Bruxism	9	15.77
		TMD	13	22.80
		Modulation	4	7.02
		**Total complaints**	**57**	**100**

**Caption:** N = total number; % = percentage

In Table 9, in the somatosensory assessment, the individuals obtained greater modulations in acupressure (masseter, pre- and post-auricular, temporal tendon, mastoid, trapezius, and spinal) and in mandibular movements (opening and lateralization with resistance).

**Table 9:** Somatosensory assessment findings

SOMATOSENSORY SUBGROUP				
	**Total number of subjects**	**Points**	**N**	**%**
Acupressure/palpation	33	Masseter	13	7.30
		Lateral pterygoid	9	5.06
		Medial pterygoid	8	4.49
		Pre- and post-auricular temporal	20	11.24
		Front	7	3.93
		Temporal tendon (TMJ)	18	10.11
		Zygomatic	6	3.37
		Digastric	5	2.80
		Buccinator	6	3.38
		Orbiculares	3	1.68
		Geniohyoid	2	1.12
		Long neck	9	5.05
		Sternocleidomastoid	6	3.37
		Mastoid	17	9.55
		Scalene	6	3.38
		Scapula lifter	5	2.80
		Rhomboid	5	2.80
		Suboccipital muscles	10	5.62
		Splenium	9	5.06
		Trapezius and spinous processes	14	7.87
		**Total modulations**	**178**	**100%**
	**Total number of subjects**	**Movements**	**N**	**%**
Mandibular movements	33	Closing eyes tightly	9	9.09
		Teeth clenching	9	9.09
		Mandibular retrusion	8	8.08
		Opening without resistance	6	6.06
		Opening with resistance	11	11.11
		Protrusion without resistance	5	5.05
		Protrusion with resistance	8	8.08
		Right lateralization without resistance	11	11.11
		Right lateralization with resistance	13	13.13
		Left lateralization without resistance	9	9.09
		Right lateralization with resistance	10	10.11
		**Total modulations**	**178**	**100**
	**Total number of subjects**	**Movements**	**N**	**%**
Cervical movements	33	Flexion	4	17.39
		Extension	5	21.74
		Rightward tilt	8	34.78
		Leftward tilt	6	26.09
		**Total modulations**	**23**	**100**
	**Total number of subjects**	**Movements**	**N**	**%**
Eye movements	33	Horizontal pursuit	6	17.65
		Vertical pursuit	7	20.59
		Lateralization to the right	6	17.65
		Left lateralization	5	14.71
		Upper verticalization	3	8.82
		Lower verticalization	4	11.76
		Convergence	3	8.82
		**Total modulations**	**34**	**100**

**Caption:** N = total number; % = percentage

## DISCUSSION

This research was developed to create a protocol and clinical application guide for tinnitus in adults and the elderly, in addition to verifying its applicability and results. Thus, it is worth noting the novelty of this study's contributions, given that the specialized literature demonstrates the need for a complete clinical evaluation to establish the etiological subgroups of tinnitus^(4)^. However, despite these recommendations, there is still no single comprehensive document covering everything that needs to be evaluated, with recommendations and guidelines on how clinical management should be carried out^(3,4)^.

The structuring of protocols in the health field is extremely important, as it enables the combination of a set of guidelines, procedures, and instructions that standardize the approach to the evaluation and treatment of patients in different situations. These are developed based on scientific evidence and best clinical practices, designed to guide professionals in the provision of care. Therefore, the creation of a structured protocol focused on initial assessment is of paramount importance in order to address the existing gap in tinnitus clinical practice.

Studies demonstrate the high incidence of association between generating agents (main etiological factor) and amplifiers (increased perception) and emphasize the possibility of etiological cofactors. The perspective is that, when subgroups are established in the assessments and their etiological mechanisms are understood, the diagnosis will be better and more appropriate for the effectiveness of rehabilitation^(4,5,14)^. Thus, the creation of this protocol aimed to understand the pathophysiological mechanisms in order to organize it in a way that measures all possible causes and aggravating factors of tinnitus in subgroups.

In stages 1, 2, and 3, numerous changes were suggested and made to the assessments. These were of paramount importance for the initial construction of the instrument and the application guide, given that structured and well-planned protocols serve as an essential roadmap for studies and treatments. Another relevant aspect was the inclusion of questions in the medical history and the completion of assessments focused on auditory-cognitive investigation and associated otological complaints, which can often be underdiagnosed^(15-17)^. The inclusion of new metabolic measurement rates and somatosensory assessments, as well as suggestions for imaging tests for vascular diagnosis, enabled a more comprehensive and assertive assessment of the different subgroups^(8,18,19)^.

Offering clinicians a choice of assessments and organization of application allows for the personalization of care to the specific needs of patients. In addition to adapting exams and treatment to individual preferences and the clinical context, this increases adherence to the rehabilitation process, improves the effectiveness of care, and provides a personalized approach. Thus, it was decided to leave the assessment suggestions to the professional's discretion. In this way, all the changes made are justified, with the aim of ensuring clarity, consistency, and integration between all the items involved, minimizing errors and omissions, and following the principles of psychometrics.

Steps 4 and 5 consisted of the final drafting of the protocol, based on feedback from the expert judges. As a result, one judge referred to the need for professional experience for clinical application and management. This is justified by the fact that although tinnitus is highly prevalent in the population and in audiology services, speech therapists are generally not trained in its assessment and management^(20)^. As a rule, undergraduate curricula and graduate programs in audiology do not provide comprehensive information, resulting in a gap in care. In this sense, patients who seek the professional services of a speech therapist therefore have no basis for being sure that they will receive evidence-based care^(6)^. Thus, the creation of the application guide is emphasized, whose objective was to facilitate the application and understanding of the results, ensuring that the instrument is implemented correctly, guaranteeing that the assessments are reliable and safe, based on scientific evidence.

To analyze the clinical applicability of the instrument, measure its effectiveness, accessibility, and ability to meet the needs of professionals and patients, steps 6 and 7 were carried out. In these steps, changes were suggested aimed at emotional and somatosensory assessment, detailing medication investigation, and reducing the length of the protocol, focused on somatosensory assessment and emotional screening.

Authors demonstrate that subjects seeking care suffer from the perception of the symptom, often presenting comorbid depression and anxiety, which are sometimes underdiagnosed^(21)^. Such statements are justified from the perspective of the network model , in which tinnitus can be considered an interactive system of these two symptoms^(22)^. Thus, emotional screening is justified in all cases, since the interaction of emotional symptoms in tinnitus patients can be considered a significant target for intervention for management, even in mild cases. In the same vein, somatosensory evaluation was maintained, but it should only be performed in individuals who present associated complaints in their medical history, especially if any of the four diagnostic criteria are positive^(11)^. This association is necessary because research has demonstrated modulation in the performance of otological tinnitus assessments, even when there is no somatosensory influence^(23)^.

Several causes may be associated with tinnitus, including hearing problems, systemic diseases, and side effects of medications. The relationship between medications and tinnitus is complex and can occur in different ways. Thus, a table was included for filling in the medications that the individual uses, as suggested by non-specialist judges. The pathophysiology focuses on factors such as ototoxicity, neurological or vascular effects. Thus, when a patient presents with tinnitus disorder, it is important to review their medication history to identify possible aggravating factors and guide the dialogue with the physician^(5)^.

The application time is extremely important to consider, since when it is adequate, it can provide greater adherence to its use, efficiency in care, reduction of stress due to demand, and a detailed and careful approach. In this sense, the judges suggested the possibility of reducing or dividing the assessments in order to minimize the application time. Given that the selected assessments guide the diagnosis and are performed, each with its specificity, it was decided to maintain them, but it should be noted that the clinician has the autonomy to apply them over two days, for example, as long as they are interpreted together^(4,5,8)^. Thus, the initial reception of the patient can be carried out at the first consultation, through the investigation of the medical history, the application of scales and questionnaires to understand the impacts of perception, and the audiological evaluation for the diagnosis of peripheral hearing acuity. At the second consultation, the evaluations can be finalized.

A pilot study measured the applicability of the instrument in a real-life collection situation, taking approximately 1 hour and 30 minutes. In the application, Table 3 shows a high prevalence of auditory and emotional subgroups, followed by metabolic and somatosensory subgroups. The specialized literature shows that the pathophysiology of tinnitus is often associated in 85 to 96% of cases with reduced peripheral hearing acuity and that there is a high incidence and correlation of emotional issues with the perception and/or catastrophization of the symptom^(24)^. Altered metabolic, endocrine, nutritional, and dyslipidemia rates are also reported^(25)^. Approximately 43% of individuals with non-pulsatile tinnitus are diagnosed with somatosensory tinnitus^(26)^. Thus, the findings corroborate the incidence found in the application of the CIP-t, demonstrating that it is capable of measuring etiological agents.

Regarding audiological characteristics, there was a higher incidence of mild sensorineural hearing loss or high-frequency hearing loss, as shown in Table 4. These findings have already been reported by other authors and are justified by the characteristics of the sample (age of participants and perception of symptoms)^(1)^. Furthermore, as the study was conducted at an audiological diagnostic center, patients seeking care already had otological complaints.

Although tinnitus is mainly a symptom of a hearing problem, such as hearing loss or exposure to high sound pressure levels, emotional and psychological factors can have a significant impact on the perception and intensity of tinnitus^(21)^. This study highlights the high incidence of anxiety and depression, which are usually associated, as shown in Tables 5 and 6. These changes are justified by changes in neural and hormonal pathways, which alter the functioning of the body's axes, causing changes in brain function and the activation of systems responsible for regulating mood, attention, and neural responsiveness, correlating with the perception and catastrophization of the symptom. The high incidence can also be explained by the post-pandemic period, during which uncertainty, fear, stress, and emotional issues became more evident^(27)^.

Studies show that laboratory changes in patients with tinnitus may vary depending on the underlying etiological factor and associated comorbidities . Some research has shown that there may be correlations with certain medical conditions that affect laboratory results related to glucose, thyroid hormone, vitamins, minerals, and/or cholesterol and triglycerides^(4,28)^. In the present study, Table 3 shows that more than 90% of the sample, among the subjects who underwent the evaluation, presented laboratory abnormalities. Of these, the highest prevalence of alterations was related to cholesterol, blood count, and vitamin D (Table 7). Thus, the suggested evaluation demonstrates the importance of laboratory measurement, enabling guidance and referral for clinical management by a specialized professional. It is also noteworthy that it minimizes unnecessary referrals, avoiding subject dissatisfaction and prolongation of the intervention process.

In the somatosensory evaluation, Tables 8 and 9 show a lower incidence of participants for this subgroup, since correlating the medical history with the evaluation allows the clinician to achieve accuracy, sensitivity, and specificity in the diagnosis^(11)^. The study showed greater complaints regarding neck/jaw pain and symptoms of temporomandibular dysfunction^(29)^, with an incidence of modulation in the temporal, masseter, temporomandibular joint (TMJ) tendon, mastoid, suboccipital, and trapezius muscles. There were also similar modulations in mandibular, cervical, and ocular movements. Therefore, it should be emphasized that these results demonstrate that combining the investigative history with the evaluations is of utmost importance for an accurate diagnosis and effective treatment of cervicocraniomandibular physical therapy.

The specialized literature strongly recommends that the diagnosis of vascular tinnitus be made by investigating the perception of the symptom and the associated vascular and cardiac clinical signs and symptoms, confirmed by imaging tests^(13)^. Typically, patients belonging to this subgroup have specific tinnitus, with pulsatile, severe manifestations and a rhythm matching the heart rate or changes in blood pressure^(13)^. Thus, individuals with vascular tinnitus are already under medical supervision or seek a doctor for diagnosis, receiving initial intervention before seeking care from other professionals, which explains their absence in the present study.

In the vascular assessment, no participants belonging to this subgroup were identified. In the medical history of the participants, no signs of severe cardiac and/or vascular conditions were found, only systemic arterial hypertension (SAH), which was controlled at the time. One individual among those evaluated obtained modulation in the four assessments. However, this individual was in the somatosensory subgroup due to the presence of pulsatile tinnitus of somatosensory origin caused by myoclonus. This highlights the importance of maintaining the assessments, given that excluding such vascular factors contributes to the differential diagnosis.

Therefore, the CIP-t was created and validated. With it, it is possible to apply a single tinnitus assessment document, which enables professionals to measure all subgroups effectively, both clinically and scientifically. The separation of groups helps to guide investigation, rehabilitation, and treatment, but these concepts must be well established due to the incidence of association of symptom generators and amplifiers. In this sense, the diagnosis and referral for treatment of etiological factors should preferably be carried out by a multidisciplinary team^(4)^. Thus, understanding this overlap and overcoming the difficulty of diagnosing the factors involved in the origin of tinnitus, the next step consists of an art: treatment^(30)^.

### Study limitations and future perspectives

A limitation of the study is the small sample size in the metabolic evaluation. Possible future studies or clinical applications include adapting the protocol for digital use through electronic forms, as a way to expand its reach and facilitate its implementation in clinical practice. It should also be noted that, despite a representative sample, there is a need for further studies, increasing the sample size, as well as application in different populations, for example, in children and adolescents.

## CONCLUSION

The Tinnitus Clinical Investigation Protocol has been finalized and its content validated, making it possible to apply it to adults and elderly individuals who perceive tinnitus. Its greater relevance and applicability in tinnitus disorder is noteworthy.
