# Supplementary material for: Clinical investigation protocol for tinnitus (CIP-t): development and validation of an instrument
Source: Codas. 2026 Apr 27;38(3):e20250125. doi: 10.1590/2317-1782/e20250125en (PMC13193187; doi:10.1590/2317-1782/e20250125en)
Supplement: Translation

# Translation: Protocolo de investigação clínica no zumbido (PIC-z): desenvolvimento e validação de um instrumento

**DOI:** 10.1590/2317-1782/e20250125pt

**Authors:** Hélinton Goulart Moreira, Michele Vargas Garcia

**Affiliations:** 1 Programa de Pós-graduação em Distúrbios da Comunicação Humana, Departamento de Fonoaudiologia, Universidade Federal de Santa Maria – UFSM - Santa Maria (RS), Brasil.

**Keywords:** Zumbido, Protocolo, Avaliação, Adultos, Idosos

## Abstract

**Objetivo:**

O objetivo do estudo foi o desenvolvimento e validação de um protocolo de investigação clínica do zumbido e analisar a sua aplicabilidade.

**Método:**

A pesquisa foi realizada em 8 etapas: Etapa 1: Revisão bibliográfica e desenvolvimento do protocolo-piloto; Etapa 2: Juízes especialistas I - primeira análise/julgamento; Etapa 3: Elaboração do protocolo único de investigação e do guia de aplicação; Etapa 4: Juízes especialistas II - segunda análise/ julgamento dos instrumentos da etapa anterior; Etapa 5: Ajustes nos materiais; Etapa 6: Juízes especialistas e não especialistas - aplicação clínica; Etapa 7: Construção final; Etapa 8: Estudo piloto - amostra por conveniência. Os resultados de cada etapa foram alocados em uma planilha do Excel para análises específicas (cálculo da razão da validade de conteúdo e sugestões).

**Resultados:**

Foi possível a estruturação do protocolo, dando origem a um instrumento único, subdividido em: anamnese de investigação do zumbido, histórico audiológico, emocional, metabólico, somatossensorial, vascular e resultados das avaliações, quadro para definição de subgrupos, árvore de decisão clínica e avaliações complementares. A aplicação foi realizada em uma amostra de 83 adultos e idosos.

**Conclusão:**

O protocolo de investigação clínica do zumbido foi finalizado e seu conteúdo validado, sendo possível aplicar em indivíduos adultos e idosos com zumbido.

## INTRODUÇÃO

O zumbido crônico é um sintoma otológico de causa multifatorial e incidência de aproximadamente 740 milhões de adultos em todo o mundo, sendo percebido como um grande problema por mais de 120 milhões de pessoas, principalmente em sujeitos com idades acima de 65 anos^(1)^.

Inicialmente, sua fisiopatologia foi explicada pelo modelo neurofisiológico, evidenciando sua origem periférica, detectada a nível de sistema nervoso auditivo central, ocasionando incômodo quando associada ao sistema límbico. Tendo em vista suas manifestações, um estudo recente sugeriu que este deve ser intitulado como transtorno quando associado a prejuízos cognitivos, de percepção de fala e/ou alterações emocionais^(2)^.

A multifatorialidade etiológica que pode desencadeá-lo e influenciá-lo torna cada sujeito único. Assim, para que o processo de intervenção seja efetivo, é necessário um programa de avaliação completa e a realização de um tratamento individualizado^(3-5)^. Nesse sentido, os gestores de saúde pública devem considerar o impacto global do zumbido, investindo em mais pesquisas e políticas públicas para impulsionar a sua investigação^(1)^.

Dada a sua alta prevalência e o impacto negativo que exerce na vida dos indivíduos, o zumbido pode ser considerado uma questão de Saúde Pública e precisa ser incluído no planejamento em todos os níveis de atenção à saúde. Outro aspecto relevante refere-se ao desconhecimento profissional, dos quais a maioria, mesmo com especialização na área de audiologia, não consideram-se aptos para a avaliação e manejo clínico nesta população^(6)^.

A falta de conhecimento dos profissionais aponta para as dificuldades que os pacientes provavelmente encontrarão para identificar os cuidados de saúde mais apropriados. Uma jornada de tratamentos incertos e a falta de caminhos claros de encaminhamento, provavelmente são fatores agravantes do sofrimento, gravidade e cronicidade do zumbido. Surgem, como possibilidades de sanar tais lacunas, a criação de protocolos, que focam nos cuidados iniciais oferecidos aos pacientes por profissionais da saúde. Estes documentos abordam temas como a prevenção, promoção da saúde, gestão de condições crônicas e encaminhamentos para especialistas, garantindo que o atendimento seja integrado e coordenado de modo efetivo.

O que justifica este estudo é a inexistência de um instrumento integrativo em língua portuguesa para investigação detalhada dos subgrupos do zumbido, buscando mensurar os diferentes fatores etiológicos geradores e amplificadores do sintoma, que na maioria das vezes estão associados. Estes podem ser auditivos, emocionais, vasculares, somatossensoriais e/ou metabólicos, e precisam estar bem definidos na avaliação clínica, para uma melhor assertividade terapêutica^(4)^. Ao tratar a causa do sintoma, aumenta a possibilidade de remissionar ou reduzir o transtorno causado pelo zumbido.

A hipótese é que, por meio de uma avaliação clínica detalhada, tem-se um tratamento mais efetivo, em decorrência da multifatorialidade etiológica do zumbido, ou seja, ao identificar os agentes do sintoma é possível a realização de um tratamento mais adequado^(5,7,8)^. Portanto, o objetivo do estudo é o desenvolvimento de um protocolo e de um guia de aplicação clínica para o zumbido, bem como analisar a sua aplicabilidade.

## MÉTODO

### Aspectos éticos

Trata-se de um estudo de caráter quali-quantitativo e transversal, aprovado pelo Comitê de Ética em Pesquisa da Universidade Federal de Santa Maria (UFSM), sob o número 57700721.0.0000.5346. Salienta-se que a pesquisa cumpriu todas as diretrizes previstas para realização de pesquisas com seres humanos vigentes na Resolução 466/12 do Conselho Nacional de Saúde do Brasil. Todos os juízes participantes e os indivíduos do estudo-piloto assinaram um Termo de Consentimento Livre e Esclarecido específico, concedendo sua participação na pesquisa.

Os participantes da aplicação do protocolo foram esclarecidos quanto aos riscos e benefícios da sua participação. Todas as avaliações foram realizadas no Ambulatório de Audiologia de uma instituição de ensino superior. Além disso, os indivíduos configurados com transtorno do zumbido foram acolhidos e receberam tratamento no ambulatório especializado de zumbido da instituição.

### Procedimentos e participantes

Para garantir uma melhor compreensão metodológica, a pesquisa foi estruturada em fases e etapas. No Quadro 1 é apresentado um cronograma acerca das fases, etapas, participantes e critérios de seleção.

**Quadro 1:** Descrição dos participantes e critérios de seleção para cada etapa do processo de criação e validação de conteúdo do Protocolo de Investigação Clínica do Zumbido (PIC-z)

CONSTRUÇÃO E VALIDAÇÃO DE CONTEÚDO			
Fases	Etapas	Participante	Critério de seleção
Fase 1	Etapa 1: Revisão bibliográfica e desenvolvimento do protocolo-piloto	02 fonoaudiólogos	Autores do estudo.
	Etapa 2: Juízes especialistas I - primeira análise/julgamento do protocolo-piloto	03 fonoaudiólogas 01 fisioterapeuta 01 médico	Doutores, com no mínimo 10 anos de atuação clínica e de pesquisa, sendo todos da área do zumbido.
	Etapa 3: Elaboração do protocolo único de investigação e do guia de aplicação.	02 fonoaudiólogos	Autores do estudo.
Fase 2	Etapa 4: Juízes especialistas II - segunda análise/ julgamento do protocolo e do guia de aplicação (mesmos da etapa 2)	03 fonoaudiólogas 01 fisioterapeuta 01 médico	Doutores, com no mínimo 10 anos de atuação clínica, sendo todos da área do zumbido.
	Etapa 5: Ajustes no protocolo e no guia de aplicação.	02 fonoaudiólogos	Autores do estudo.
	Etapa 6: Juízes especialistas e não especialistas - aplicação clínica	Especialistas: 04 fonoaudiólogos 01 fisioterapeuta Não especialistas: 04 estudantes de fonoaudiologia 01 medicina	Profissionais com no mínimo 02 anos de atuação clínica na área da audiologia ou zumbido. Discentes com experiência com zumbido menor que um ano.
	Etapa 7: Construção final do PIC-z	02 fonoaudiólogos	Autores do estudo.
Fase 3	Etapa 8: Estudo piloto - amostra por conveniência	83 indivíduos	Adultos e idosos, com idades entre 17 a 80 anos, com percepção de zumbido.

**Legenda:** PIC-z = Protocolo de Investigação Clínica do zumbido

A seguir, as etapas metodológicas estão descritas minuciosamente, conforme os preceitos psicométricos, relacionadas à construção e validação de conteúdo.

### Fase 1 – Desenvolvimento do protocolo e elaboração de um guia de aplicação

Nesta fase, foi elaborado um protocolo para profissionais da saúde, integrando diversos instrumentos de investigação do zumbido. Compuseram a fase as etapas 1, 2 e 3 do processo de construção e validação de conteúdo.

Na etapa 1 foi realizada uma revisão bibliográfica pelos autores da pesquisa para o desenvolvimento de um protocolo-piloto com as avaliações sugeridas. Este protocolo, junto a um questionário de julgamento, ambos elaborados pelos autores, foram enviados a cinco juízes especialistas (três fonoaudiólogos, um fisioterapeuta e um médico), doutores e com no mínimo dez anos de experiência clínica e de pesquisa na área do zumbido selecionados para realização do julgamento inicial.

A escolha dos juízes foi baseada considerando a experiência na área de elaboração do instrumento, bem como no conhecimento prévio dos processos metodológicos envolvidos na construção de novas ferramentas^(9)^.

A etapa 2 consistiu na primeira análise realizada pelos juízes especialistas. Após envio de suas respostas aos autores do estudo, as considerações foram tabuladas em uma planilha *Excel* e posteriormente foi realizado o cálculo da Razão de Validade de Conteúdo (RVC). A análise da RVC, conforme proposto por Lawshe^(9)^, considera o grau de concordância entre os avaliadores em relação à relevância de cada item do instrumento. Os itens são categorizados em três níveis: essencial, útil mas não essencial, e não essencial. Para o cálculo do RVC, utilizou-se a quantidade de juízes que classificaram o item como essencial (ne) e o total de avaliadores envolvidos na análise (N), aplicando-se a seguinte fórmula: RVC = (ne – N/2) / (N/2). Destaca-se que o cálculo RVC foi considerado adequado, para todas as etapas, quando obtivesse valores de ≥ 0,99, conforme o número de juízes participantes^(9)^.

A partir de tais análises, os autores do estudo criaram a primeira versão do protocolo em formato único e do guia de aplicação, constituindo esta a etapa 3.

### Fase 2 – Validação do protocolo por especialistas

O protocolo foi validado por meio da análise de juízes especialistas, juízes não especialistas e autores. Esta fase abrangeu as etapas 4, 5, 6 e 7 do processo metodológico.

A etapa 4 consistiu na segunda análise do protocolo e do guia de aplicação, realizada pelos mesmos juízes especialistas da etapa 2. Já a etapa 5 foi realizada pelos autores do estudo, dos quais um novo cálculo RVC foi mensurado e analisadas as sugestões dos juízes, sendo essas utilizadas para os ajustes iniciais em ambos documentos (protocolo e guia de aplicação).

Posteriormente aos ajustes, deu-se início à etapa 6, por meio da aplicação clínica do protocolo estruturado e análise do guia de aplicação. Esta etapa, foi realizada por 5 juízes especialistas (quatro fonoaudiólogos e uma fisioterapeuta, com expertise de no mínimo dois anos com zumbido) e 5 não especialistas (quatro discentes do curso de fonoaudiologia e um do curso de medicina, com experiência superior a três meses e menor que um ano com indivíduos com zumbido), objetivando mensurar a aplicabilidade clínica do instrumento.

Por fim, a etapa 7 foi realizada pelos autores do estudo, por meio dos ajustes e construção final do PIC-z e do Guia de Aplicação.

### Fase 3 – Aplicabilidade do protocolo em uma amostra

Nesta etapa, correspondente à etapa 8, foi realizada a validação do protocolo com uma amostra por conveniência, utilizando o método observacional transversal. Desse modo, participaram do estudo os indivíduos que buscaram atendimento na clínica escola da instituição, com percepção de zumbido uni ou bilateral, de diferentes tempos (crônicos ou não), percepções, locais e manifestações, com o objetivo de analisar a aplicabilidade do instrumento em uma situação real de coleta.

O Protocolo de Investigação Clínica no Zumbido (PIC-z) foi aplicado pelos autores do estudo e pelos membros do Grupo de Pesquisa no Serviço de Atendimento Fonoaudiológico- UFSM, posteriormente à realização de treinamento. A administração do instrumento foi realizada em uma única sala com cabine acusticamente tratada e espaço físico suficientemente grande. O diagnóstico audiológico foi realizado por meio dos seguintes equipamentos: otoscópio modelo TK, marca *Mikatos*, fones de ouvido supra-aurais (*Telephonics* TDH-39), audiômetro modelo AD629B e o imitanciometro AT235, ambos da marca *Interacoustics*. Ainda, no mesmo ambiente, foram realizadas as avaliações e a aplicação dos questionários, dos quais para a avaliação somato e vascular os indivíduos foram sentados em uma cadeira confortável e reclinável. Após a aplicação, os dados foram alocados em uma planilha do *Excel* para as posteriores análises acerca dos achados do protocolo.

O PIC-z foi aplicado em uma amostra-piloto de 83 adultos e idosos de ambos os sexos (55 mulheres e 28 homens), com idades de 17 a 80 anos (média-idade= 51,77 anos; desvio-padrão=26,35) e com tempo de percepção que variou de um a 600 meses (média-meses=91,42; desvio-padrão=105,64). Desses, 73 apresentaram manifestações constantes e 10 intermitentes. Ainda, dentre os indivíduos avaliados, um relatou percepção aguda (menos de três meses), 74 cronicidade e oito não souberam dizer o início da percepção do sintoma.

Na Tabela 1, torna-se possível observar as manifestações e características referentes ao zumbido.

**Tabela 1:** Características da amostra referentes ao zumbido (N=83)

Categoria	Descrição	N (total de sujeitos)
Localização	Cabeça	9
	Orelha direita	7
	Orelha esquerda	12
	Bilateral	11
	Ambas, pior a direita	30
	Ambas, pior a esquerda	14
Tipo de percepção	Única	71
	Múltipla	12
Percepções Únicas (n=71)	Apito	23
	Cigarra / Grilo**	12 (cada)
	Abelha	6
	Chiado / Panela de pressão**	4 (cada)
	Cachoeira	2
	Ambulância / asa de abelha / coração / motor / sopro / trator / turbina de jato / ventania**	1 (cada)
Percepções múltiplas (n=12)	Cigarra + apito	4
	Apito + grilo	3
	Grilo + Cigarra	2
	Apito + panela de pressão	1
	Cachoeira + Abelha	1
	Cachoeira + Grilo	1
Frequência tonal do zumbido	Aguda	57
	Média	16
	Grave	10

** Foram acoplados devido apresentarem a mesma incidência (exemplo= cigarra / grilo obtiveram-se 12 cada, totalizando 24 sujeitos)

Na Tabela 2, observa-se o incômodo, volume e grau do zumbido entre os participantes, dos quais apresentaram-se moderados.

**Tabela 2:** Descrição do incômodo, volume e grau do zumbido entre os participantes (N=83)

Medida	Média	Mínima	Máxima	Desvio-padrão
EVA- Incômodo	6.84	2	10	3,56
EVA- Volume	6.49	2	10	3,41
THI- total	46.77	10	100	21,73
THI- funcional	19.90	4	44	12,59
THI - emocional	17.24	0	38	11,22
THI-catastrófico	10.80	0	40	7,48

**Legenda:** EVA = escala visual analógica; THI = *tinnitus handicap inventory*

Destaca-se que todos os participantes conseguiram realizar as avaliações, sem demonstrar cansaço, apenas desconforto na realização da digito-pressão. O protocolo foi aplicado e finalizado com tempo médio de aproximadamente uma hora e trinta minutos. Portanto, os itens do instrumento foram suficientes para contemplar o objetivo da avaliação.

## RESULTADOS

### Etapa 1 – Revisão bibliográfica e desenvolvimento do protocolo-piloto

Nesta etapa, foram utilizados três estudos: Tunkel et al.^(3)^, Onishi et al.^(4)^ e Cima et al.^(8)^, selecionados devido sua abrangência nas avaliações e relevância científica. As avaliações escolhidas encontram-se descritas abaixo.

- História clínica: Utilizada para o processo inicial do diagnóstico e intervenção. Desse modo, foi selecionada a anamnese de investigação do zumbido.
- Questionários e escalas: Necessárias para a compreensão e distinção da percepção de zumbido para o transtorno do zumbido. Assim, foi selecionado o *Tinnitus Handicap Inventory* (THI) - repercussão do zumbido na qualidade de vida (grau, impactos funcionais, emocionais e catastróficos) e a Escala Visual Analógica (EVA) - intensidade e volume do sintoma.
- Avaliação audiológica: Utilizada no diagnóstico de alterações auditivas, orientações e intervenção. Foram selecionadas a inspeção visual do meato acústico externo, audiometria tonal liminar (250 a 8000Hz), logoaudiometria (Limiar de reconhecimento de fala e índice percentual de reconhecimento de fala) e medidas de imitância acústica (imitanciometria e reflexos acústicos estapedianos contralaterais).

As avaliações a seguir são recomendadas para investigar fatores amplificadores predisponentes ou coadjuvantes na percepção do zumbido. É importante destacar que, embora a triagem emocional, avaliação vascular, somatossensorial ou a solicitação de exames laboratoriais possam ser realizadas pelo fonoaudiólogo a fim de estabelecerem-se subgrupos referentes a percepção, elas não serão conduzidas por tal profissional (CFFa nº 246/2000)^(10)^. O objetivo principal é identificar os fatores amplificadores, possibilitando encaminhamentos precisos.

- Avaliação somatossensorial: Deve ser realizada por um profissional capacitado e treinado, devido ao tempo e força na aplicação de cada técnica. É utilizada para a investigação das influências somáticas sobre a percepção do zumbido, relacionadas às regiões da cabeça, face e do pescoço. Foram selecionadas como possibilidade de realização, quando associadas aos quatro critérios diagnósticos propostos por Michiels^(11)^, às seguintes avaliações: modulação do dígito-pressão/palpação, manobra intra-conduto, movimentos mandibulares, movimentos cervicais e movimentos oculares^(11)^.
- Triagem emocional: Pode ser aplicada por qualquer profissional da saúde e investiga fatores emocionais, principalmente, a ansiedade e depressão como amplificadores e agravadores do zumbido. Para contemplar o objetivo, foi selecionada a Escala Hospitalar de Ansiedade e Depressão (EHAD)^(12)^.
- Exames laboratoriais: Foi selecionada a investigação das seguintes taxas: Hemograma completo, Glicemia de jejum, Hemoglobina glicada, Colesterol total e frações, Triglicerídeos, Glicose, Insulina, T4 livre, TSH, Zinco, Magnésio, Vitamina B12, Vitamina B9, Cortisol, Serotonina, Vitamina D, Vitamina A, Vitamina C, Ferro, Ferritina, Cálcio, que podem ser fatores etiológicos, predisponentes ou coadjuvantes^(4)^.
- Triagem vascular: Utilizada para correlação de possíveis alterações vasculares e cardíacas ao zumbido pulsátil, como malformações, fístulas arteriovenosas, hipertensão intracraniana e mudanças de fluxo, que podem ocasionar o zumbido pulsátil. Assim, foram selecionadas a manobra de compressão da artéria carótida e lateralização de cabeça para ambos os lados^(13)^. O diagnóstico de alterações vasculares é realizado pelo médico e a triagem tem apenas a função de direcionar o paciente.
- Avaliações audiológicas altamente recomendadas: foram sugeridas para investigar fatores etiológicos não evidenciados na avaliação audiológica básica. Apresenta por objetivo mensurar as demandas do indivíduo ou analisar as queixas autorrelatadas. Desse modo, foram selecionadas:
- Avaliações psicoacústicas do zumbido (acufenometria- frequência e intensidade, nível mínimo de mascaramento e inibição residual). Estas mensurações podem ser utilizadas para o conhecimento do paciente sobre seu sintoma, no aconselhamento, no acompanhamento dos tratamentos e na indicação de tratamentos relacionados à terapia sonora.
- Audiometria de altas frequências - 9000 a 20.000Hz e/ou Emissões Otoacústicas Transientes e/ou por Produto de Distorção, mediante normalidade na avaliação audiológica convencional.
- Limiar de desconforto auditivo- 250 a 8000Hz com tom puro e fala para aqueles sujeitos com queixa de desconforto aos sons.
- Teste de Função Tubária e questionário de autopercepção para indivíduos com queixas de plenitude auricular.
- Potenciais evocados auditivos de tronco encefálico e longa latência para diagnóstico diferencial e compreensão das mudanças neurofisiológicas advindas do sintoma.

Diante das recomendações nacionais e internacionais, o protocolo-piloto foi elaborado, com base na revisão realizada. Foi feito um compilado em um documento *Word* dos protocolos e avaliações, com orientações de realização, e enviado para análise de juízes.

### Etapas 2 e 3 – Juízes especialistas I (primeira análise) e elaboração do Protocolo Único de Investigação e do Guia de Aplicação

De modo geral, na etapa 2, foram necessárias mudanças em todas as avaliações sugeridas no protocolo-piloto e enviadas aos juízes especialistas.

Na anamnese, evidenciou-se um RVC de 0.2, sendo sugeridas mudanças referentes à inserção de dados pessoais, separação do protocolo em tópicos de investigação e inclusão de questionamentos acerca do zumbido, transtornos da percepção do sons e sobre a síndrome tônica do tensor do tímpano.

Nos questionários e escalas, observou-se um RVC de 0.6 sendo apenas sugerido a inclusão de um questionário que avalia a qualidade de vida em sujeitos com zumbido por apresentar mais domínios.

Em relação à avaliação audiológica, foi recomendado a inclusão da avaliação das habilidades auditivas e cognitivas, do limiar de desconforto, inibição residual e a criação de um espaço destinado aos resultados audiológicos pregressos, demonstrando um RVC de 0.4.

Para triagem emocional, foi sugerido a inclusão de mais dois questionários como possibilidades de aplicação (RVC= 0.8).

Na avaliação somatossensorial, observou-se um RVC de 0.6, devido à necessidade de inclusão de avaliações no exame físico, acréscimo de regiões musculares, retirar a manobra-intraconduto e a sugestão de que a avaliação somatossensorial seja realizada somente pelo profissional fisioterapeuta.

Por fim, para ambas as avaliações, metabólica e vascular, evidenciou-se um RVC de 0.4, dos quais os juízes sugeriram a inclusão de solicitação de novas taxas laboratoriais, de exames de imagem complementares e foi sendo recomendado, por um juíz, que estas sejam solicitadas apenas pelo profissional médico.

Nos questionamentos acerca da aplicabilidade do protocolo, voltado à mensuração dos subgrupos, obteve-se um cálculo RVC de 0.6, devido à sugestão de que a avaliação somatossensorial seja feita pela fisioterapeuta e a vascular e metabólica pelo profissional médico. Porém, no manejo profissional, foi de 0.8, devido à avaliação no transtorno do zumbido ser realizada de maneira multiprofissional. Em relação ao desfecho clínico para aplicação do protocolo, para inserção na prática clínica, obteve-se um RVC com concordância de 100%, sem sugestões (subgrupos auditivo e emocional). Por fim, tendo em vista a maioridade de juízes que aceitaram tais triagens, mesmo não atingindo o RVC esperado para a vascular, metabólica e somatossensorial, mantiveram-se as mesmas no protocolo.

Na etapa 3 deu-se origem à elaboração do instrumento em formato único. O mesmo foi subdividido em: identificação e informações gerais, parte I: anamnese de investigação, parte II: resultados das avaliações e parte III: subgrupos etiológicos e árvore de decisão. Sua estruturação foi realizada desta forma para que o avaliador possa ter acesso e registro às informações pessoais do paciente, como também as perguntas que devem ser realizadas durante a investigação, espaço para assinalar as queixas para cada subgrupo, bem como a descrição das avaliações que devem ser realizadas em todos os indivíduos e as altamente recomendadas mediante as queixas apresentadas.

Na parte III do protocolo, foi criado um quadro dos agentes etiológicos da percepção, com os geradores e amplificadores do sintoma. O objetivo foi que esse possa contribuir para a avaliação clínica, ou seja, que por meio do quadro o aplicador consiga compreender os subgrupos e se há necessidade de uma avaliação especializada. Deste modo, a opção “provável” refere-se à certeza da influência de determinado subgrupo, o “pouco provável” ao ter a necessidade de uma avaliação por profissional específico e o “improvável” ao ter certeza que não há influência. Ainda, foi elaborada a árvore de decisão, das quais por meio da avaliação, orienta ao avaliador o profissional mais adequado ao manejo clínico.

Tendo em vista a complexidade das avaliações e as sugestões dos juízes especialistas, optou-se por realizar um Guia de Aplicação. Esse instrumento foi elaborado para contribuir com a maneira de preenchimento, aplicação do protocolo e interpretação das avaliações por conter as orientações específicas, os instrumentos necessários e a correlação dos achados clínicos.

### Etapas 4 e 5 – Juízes especialistas II (segunda análise) e ajustes no PIC-z e Guia de Aplicação

Nesta etapa, não foram realizadas sugestões de mudanças no protocolo, demonstrando índice do RVC de 100% entre os juízes. Na questão acerca do manejo e encaminhamento, evidenciou RVC de 0.8, devido a um juiz referir que a aplicação e o manejo clínico dependerão da expertise do clínico. Diante disso, não foram realizados ajustes no protocolo.

### Etapa 6 – Juízes especialistas e não especialistas - aplicação clínica e Etapa 7 – Ajustes finais no protocolo de aplicação

Nesta etapa de aplicação, foram sugeridas pelos juízes especialistas e não especialistas mudanças pontuais no protocolo das quais encontram-se descritas e configuraram a etapa 6.

Em relação aos juízes especialistas para clareza dos itens, facilidade de aplicação, inserção na prática clínica, diagnóstico de subgrupos e encaminhamentos, obteve-se um RVC de 100%, sem sugestões.

Para o tempo de aplicação, foi de 0.6, sugerindo que a triagem emocional e somatossensorial sejam realizadas apenas em sujeitos com sinais clínicos ou que seja elaborada uma versão resumida. Para a aplicação profissional, foi de 0.2, devido à necessidade do fonoaudiológico estar sempre na equipe (avaliação audiológica), à realização de avaliações específicas para determinado profissional e à necessidade de expertise clínica para aplicação e interpretação. Do mesmo modo para o questionamento acerca da formação prévia à aplicação obteve-se um RVC de 0.8.

No espaço destinado a sugestões, os juízes recomendaram acréscimos pontuais, como investigar com maior detalhamento o tipo de exercício praticado, se o zumbido muda com o esforço físico, o tempo de jejum e inclusão da opção sem modulação na avaliação somatossensorial.

Em relação aos juízes não especialistas referente a clareza dos itens, inserção na prática clínica, formação prévia para aplicação, diagnóstico dos subgrupos e encaminhamentos, não houve sugestões, com RVC de 100% de concordância. Para o tempo de aplicação foi de 0.6, sugerindo que reduzam-se os pontos da avaliação muscular ou que seja aplicado em duas sessões. Na facilidade de aplicação obteve-se de 0.8, enfatizando que, para facilitar, sejam inseridos os pontos na avaliação somatossensorial em formato de imagem. Na aplicação multiprofissional, o RVC foi de 0.4, devido à necessidade de expertise clínica e o fonoaudiólogo estar sempre inserido na equipe. Para o espaço destinado às sugestões foi sugerida a inclusão dos medicamentos utilizados e da mudança qualitativa na percepção na avaliação somatossensorial.

Após análise e mudanças, realizadas pelos autores do estudo, deu origem à etapa 7, que consiste na finalização da versão do conteúdo, aparência e do guia de aplicação disponíveis na Figura 1.

**Figura 1:** QR Code para acesso do Protocolo de Investigação Clínica do Zumbido (PICz) e Guia de Aplicação

### Etapa 8 – Estudo piloto

Em relação aos achados da aplicação do protocolo, na Tabela 3 torna-se possível observar a incidência dos agentes etiológicos, evidenciando uma maior porcentagem para o subgrupo auditivo, emocional, somatossensorial e metabólico.

**Tabela 3:** Caracterização dos participantes considerando a amostra total, para os diferentes subgrupos

**SUBGRUPOS**				
	**N total de sujeitos**	**Limiares até 8kHz**	**N**	**%**
AUDITIVO	83	Normal	13	15,66%
		Alterada	70	84,34%
	**N total de sujeitos**	**Triagem Emocional**	**N**	**%**
EMOCIONAL	83	Normal	12	14,46%
		Alterada	71	85,54%
	**N total de sujeitos**	**Exames laboratoriais**	**N**	**%**
METABÓLICO	13	Normal	1	7,70%
		Alterado	12	92,30%
	**N total de sujeitos**	**Influência muscular**	**N**	**%**
SOMATOSSENSORIAL	83	Não	50	60,24%
		Sim	33	39,76%
	**N total sujeitos**	**Influência vascular**	**N**	**%**
VASCULAR	83	Não	83	100%
		Sim	0	0%

**Legenda:** kHz = mil hertz; N = número de participantes; % = porcentagem

Na Tabela 4 é observado os achados da avaliação audiológica, com maior incidência da perda auditiva em ambas as orelhas, do tipo sensorioneural e de grau leve ou em frequências agudas (a partir de 3KHz).

**Tabela 4:** Achados da avaliação do subgrupo auditivo

**SUBGRUPO AUDITIVO**				
	**N total de sujeitos**	**Orelhas**	**N**	**%**
Localização	70	OD	3	4,29%
		OE	2	2,86%
		AO	65	92,85%
	**N total de orelhas**	**Tipo**	**N**	**%**
Orelha direita	68	Sensorioneural	37	54,41%
		Condutiva	0	0%
		Mista	5	7,35%
		Frequências	28	38,24%%
Orelha esquerda	67	Sensorioneural	39	58,21%
		Condutiva	2	2,99%
		Mista	5	7,46%
		Frequências	21	31,34%
	**N total de orelhas**	**Grau** *	**N**	**%**
Orelha direita	68	Leve	27	67,50%
		Moderado	5	12,50%
		Moderadamente severo	4	10,00%
		Severo	1	2,50%
		Profundo	2	5,00%
		Completo/surdo	1	2,50%
		**N total de alterações**	**40**	**100%**
	**N total de orelhas**	Grau*	**N**	**%**
Orelha esquerda	67	Leve	29	67,44%
		Moderada	7	16,28%
		Moderadamente severo	3	6,98%
		Severo	4	9,30%
		Profundo	0	0%
		Completo/surdo	0	0%
		**N total de alterações**	**43**	**100%**
	**N total orelhas**	**Frequências (Hz)**	**N**	**%**
Orelha direita	68	250	9	10,34%
		500	7	8,04%
		1000	0	0%
		2000	9	10,34%
		3000	11	12,64%
		4000	17	19,55%
		6000	18	20,67%
		8000	16	18,39%
		**N total de alterações**	**87**	**100%**
	**N total de orelhas**	**Frequências (Hz)**	**N**	**%**
Orelha esquerda	67	250	4	5,00%
		500	3	3,75%
		1000	0	0%
		2000	3	3,75%
		3000	14	17,50%
		4000	19	23,75%
		6000	19	23,75%
		8000	18	22,50%
		**N total de alterações**	**80**	**100%**

* Para esta análise foi utilizado os sujeitos que foram classificados para o grau de acordo com a média quadritonal (500, 1000, 2000 e 4000Hz)

**Legenda:** OD = orelha direita; OE = orelha esquerda; AO = ambas as orelhas; Hz = Hertz; N = número total; %=porcentagem

Em relação às queixas emocionais descritas na Tabela 5, evidencia-se a presença de ansiedade e depressão, seguidos pelo estresse.

**Tabela 5:** Queixas autorrelatadas para o subgrupo emocional

**SUBGRUPO EMOCIONAL**				
	**N total de sujeitos**	**Queixas**	**N**	**%**
Anamnese	71	Sem queixas	17	16,50%
		Ansiedade	44	42,72%
		Estresse	14	13,59%
		Depressão	19	18,45%
		Pânico/medo/fobia	8	7,77%
		Outro	1	0,97%
		**N total de queixas**	**103**	**100%**

**Legenda:** N = número total; % = porcentagem

Na Tabela 6, por meio da avaliação emocional, é demonstrada a presença de ansiedade e depressão, com um escore moderado.

**Tabela 6:** Achados da avaliação emocional

Escala Hospitalar de Ansiedade e Depressão (EHAD)				
Análises	N total	Média	Mínima- Máxima	Desvio-padrão
Total	71	15,28	1 -30	9,35
Ansiedade		8,42	0 - 16	5,00
Depressão		6,86	0 - 17	4,87

**Legenda:** N = número total

Na Tabela 7, nos achados da mensuração laboratorial, houve incidência de taxas alteradas para hemograma, colesterol e vitamina D.

**Tabela 7:** Achados da avaliação metabólica

**SUBGRUPO METABÓLICO**				
	**N total de sujeitos**	**Exames laboratoriais**	**N**	**%**
Taxas laboratoriais	12	Hemograma	3	12%
		Glicemia	2	8%
		Colesterol	5	20%
		Triglicerídeos	2	8%
		Glicose	2	8%
		TSH	2	8%
		Cortisol	1	4%
		Ferro	1	4%
		Ferritina	1	4%
		Vitamina C	1	4%
		Vitamina D	4	16%
		Zinco	1	4%
		**Total de Taxas**	**25**	**100%**

**Legenda:** N = número total; % = porcentagem

Em relação às queixas osteomusculares autorrelatadas evidenciadas na Tabela 8, houve uma maior incidência de dor e disfunção temporomandibular.

**Tabela 8:** Queixas osteomusculares autorrelatadas do subgrupo somatossensorial

**SUBGRUPO SOMATOSSENSORIAL**				
	**N total de sujeitos**	**Queixas**	**N**	**%**
Anamnese	**33**	Dor	23	40,35%
		Tensão	8	14,04%
		Bruxismo	9	15,77%
		DTM	13	22,80%
		Modulação	4	7,02%
		**Total de queixas**	**57**	**100%**

**Legenda:** N = número total; % = porcentagem

Na Tabela 9, na realização da avaliação somatossensorial, os indivíduos obtiveram maiores modulações na digitopressão (masseter, pré e pós auricular, tendão do temporal, mastoide, trapézio e espinhal) e nos movimentos mandibulares (abertura e lateralização com resistência).

**Tabela 9:** Achados da avaliação somatossensorial

**SUBGRUPO SOMATOSSENSORIAL**				
	**N total de sujeitos**	**Pontos**	**N**	**%**
Digitopressão/palpação	33	Masseter	13	7,30%
		Pterigóideo lateral	9	5,06%
		Pterigóideo medial	8	4,49%
		Temporal pré e pós auricular	20	11,24%
		Frontal	7	3,93%
		Tendão temporal (ATM)	18	10,11%
		Zigomático	6	3,37%
		Digástrico	5	2,80%
		Bucinador	6	3,38%
		Orbiculares	3	1,68%
		Gênio-hióideo	2	1,12%
		Longo do pescoço	9	5,05%
		Esternocleidomastoideo	6	3,37%
		Mastóide	17	9,55%
		Escaleno	6	3,38%
		Levantador da escápula	5	2,80%
		Rombóide	5	2,80%
		Suboccipitais	10	5,62%
		Esplênios	9	5,06%
		Trapézio e espinhas	14	7,87%
		**Total de modulações**	**178**	**100%**
	**N total de sujeitos**	**Movimentos**	**N**	**%**
Movimentos mandibulares	33	Fechar os olhos com força	9	9,09%
		Apertamento dentário	9	9,09%
		Retrusão mandibular	8	8,08%
		Abertura sem resistência	6	6,06%
		Abertura com resistência	11	11,11%
		Protrusão sem resistência	5	5,05%
		Protrusão com resistência	8	8,08%
		Lateralização direita sem resistência	11	11,11%
		Lateralização direita com resistência	13	13,13%
		Lateralização esquerda sem resistência	9	9,09%
		Lateralização direita com resistência	10	10,11%
		**Total de modulações**	**178**	**100%**
	**N total de sujeitos**	**Movimentos**	**N**	**%**
Movimentos cervicais	33	Flexão	4	17,39%
		Extensão	5	21,74%
		Inclinação para direita	8	34,78%
		Inclinação para esquerda	6	26,09%
		**Total de modulações**	**23**	**100%**
	**N total de sujeitos**	**Movimentos**	**N**	**%**
Movimentos oculares	33	Perseguição horizontal	6	17,65%
		Perseguição vertical	7	20,59%
		Lateralização para direita	6	17,65%
		Lateralização para esquerda	5	14,71%
		Verticalização superior	3	8,82%
		Verticalização inferior	4	11,76%
		Convergência	3	8,82%
		**Total de modulações**	**34**	**100%**

**Legenda:** N = número total; % = porcentagem

## DISCUSSÃO

A presente pesquisa foi desenvolvida para elaborar um protocolo e um guia de aplicação clínica do zumbido para o público adulto e idoso, além de verificar a sua aplicabilidade e os resultados. Desse modo, cabe salientar o ineditismo das contribuições deste estudo, tendo em vista que a literatura especializada demonstra a necessidade de uma avaliação clínica completa, objetivando estabelecer os subgrupos etiológicos do zumbido^(4)^. No entanto, apesar de tais recomendações, ainda não se tem um único documento abrangente com tudo que precisa ser avaliado, com as recomendações e orientações com a maneira como o manejo clínico deve ser realizado^(3,4)^.

A estruturação de protocolos na área da saúde é de extrema relevância, pois possibilita a junção de um conjunto de diretrizes, procedimentos e instruções que padronizam a abordagem de avaliação e o tratamento dos pacientes em diferentes situações. Esses são desenvolvidos com base em evidências científicas e nas melhores práticas clínicas, projetados para orientar profissionais na prestação de cuidados. Portanto, a criação de um protocolo estruturado voltado à avaliação inicial torna-se de suma importância a fim de sanar a lacuna existente na clínica do zumbido.

Estudos demonstram a grande incidência da associação entre os agentes geradores (principal fator etiológico) somados a amplificadores (aumento da percepção) e enfatizam a possibilidade de co-fatores etiológicos. A perspectiva é que, quando se estabelecer os subgrupos nas avaliações e compreender seus mecanismos etiológicos, melhor será o diagnóstico e mais adequada à efetividade da reabilitação^(4,5,14)^. Desse modo, a criação do presente protocolo objetivou compreender os mecanismos fisiopatológicos, a fim de organizá-lo de maneira que mensure todos os possíveis causadores e agravantes do zumbido, em subgrupos.

Nas etapas 1, 2 e 3, foram sugeridas e realizadas inúmeras mudanças nas avaliações. Essas foram de suma importância para a construção inicial do instrumento e do guia de aplicação, tendo em vista que protocolos estruturados e bem planejados servem como um roteiro essencial para estudos e tratamentos. Outro aspecto relevante foi a inclusão de questionamentos na anamnese e a complementação das avaliações, voltadas à investigação auditiva-cognitiva e queixas otológicas associadas, que podem muitas vezes serem subdiagnosticadas^(15-17)^. A inserção de novas taxas de mensurações metabólicas e avaliações somatossensoriais, bem como as sugestões de exames de imagem para diagnóstico vascular, possibilitaram uma avaliação mais abrangente e assertiva dos diferentes subgrupos^(8,18,19)^.

Oferecer ao clínico a escolha de avaliações e organização de aplicação possibilita a personalização do atendimento às necessidades específicas dos pacientes. Além de adaptar os exames e tratamento às preferências individuais e ao contexto clínico, aumentando a aderência ao processo de reabilitação, melhorando a eficácia dos cuidados e proporcionando uma abordagem personalizada. Assim, optou-se por deixar as sugestões de avaliações a critério profissional. Desse modo, justificam-se todas as modificações realizadas, objetivando que estas, portanto, assegurem a clareza, consistência e integração entre todos os itens envolvidos, minimizando erros e omissões e seguindo os princípios de psicometria.

As etapas 4 e 5 consistiram na elaboração final do protocolo, por meio do retorno dos juízes especialistas. Como resultados, um juiz referiu a necessidade da experiência profissional para aplicação e manejo clínico. Tal fato, é justificado que embora o zumbido seja altamente prevalente na população e nos serviços de audiologia, os fonoaudiólogos geralmente não são treinados para sua avaliação e gerenciamento^(20)^. A base curricular na graduação e os programas de pós-graduação em audiologia, como regra, não fornecem informações abrangentes, ocasionando uma defasagem no atendimento. Nesse sentido, os pacientes que buscam os serviços profissionais de um fonoaudiólogo, portanto, não têm base para ter certeza de que receberão cuidados baseados em evidências^(6)^. Salienta-se, assim, a criação do guia de aplicação, cujo objetivo foi de ser um facilitador na aplicação e compreensão dos resultados, assegurando que o instrumento seja implementado corretamente, garantindo que as avaliações sejam confiáveis e seguras, pautadas nas evidências científicas.

Para analisar a aplicabilidade clínica do instrumento, mensurar a sua eficácia, a acessibilidade e a capacidade de atender às necessidades dos profissionais e pacientes, realizaram-se as etapas 6 e 7. Nessas, foram sugeridas mudanças voltadas à avaliação emocional e somatossensorial, detalhamento da investigação medicamentosa e redução da extensão do protocolo, voltados à avaliação somatossensorial e triagem emocional.

Autores demonstram que os sujeitos que buscam atendimento sofrem com a percepção do sintoma, apresentando frequentemente depressão e ansiedade comórbidas, por vezes subdiagnosticadas^(21)^. Tais afirmações são justificadas pela perspectiva do modelo de rede, do qual o zumbido pode ser considerado um sistema interativo desses dois sintomas^(22)^. Assim, é justificada a permanência da triagem emocional em todos os casos, já que a interação dos sintomas emocionais em portadores de zumbido podem ser considerados um alvo significativo de intervenção para o gerenciamento mesmo que em quadros leves. Na mesma prerrogativa, manteve-se a avaliação somatossensorial, porém, esta deve ser realizada apenas em indivíduos que apresentam queixas associadas na anamnese, principalmente quanto positivos algum dos quatro critérios diagnósticos^(11)^. Torna-se necessária tal associação, em decorrência de pesquisas demonstrarem a modulação na realização das avaliações no zumbido otológico, mesmo este não tendo influência somatossensorial^(23)^.

Diversas causas podem estar associadas ao zumbido, incluindo problemas auditivos, doenças sistêmicas, e efeitos colaterais de medicações. A relação entre medicações e zumbido é complexa e pode ocorrer de diferentes maneiras. Desse modo, foi incluído um quadro para preenchimento das medicações que o indivíduo faz uso, como sugerido pelos juízes não especialistas. A fisiopatologia centra-se em fatores como a ototoxicidade, efeitos neurológicos ou vasculares. Assim, quando um paciente apresenta transtorno do zumbido, é importante revisar seu histórico de medicamentos para identificar possíveis agravantes e orientar o diálogo com o médico^(5)^.

O tempo de aplicação é de extrema relevância a ser considerado, já que ao ser adequado pode proporcionar uma maior adesão ao seu uso, eficiência no atendimento, redução do estresse pela demanda e uma abordagem com detalhamento e cuidado. Nesse sentido, foi sugerido pelos juízes a possibilidade de redução ou divisão das avaliações, a fim de minimizar o tempo de aplicação. Tendo em vista que as avaliações selecionadas conduzem o diagnóstico e são realizadas, cada uma com sua especificidade, optou-se por mantê-las, mas salienta-se que o clínico possui autonomia para aplicação em dois dias, por exemplo, desde que essas sejam interpretadas em conjunto^(4,5,8)^. Deste modo, pode ser realizado na primeira consulta o acolhimento inicial ao paciente, por meio da anamnese de investigação, a aplicação das escalas e questionários para compreender os impactos da percepção e a avaliação audiológica para diagnóstico da acuidade auditiva periférica. Já na segunda consulta as avaliações podem ser finalizadas.

Foi mensurada, por meio de um estudo-piloto, a aplicabilidade do instrumento em uma situação real de coleta, perfazendo aproximadamente 1 hora e 30 minutos. Na aplicação, foram evidenciados na Tabela 3 uma alta prevalência dos subgrupos auditivos e emocionais, seguidos da metabólica e somatossensorial. A literatura especializada demonstra que a fisiopatologia do zumbido está frequentemente associada em 85 a 96% dos casos com a redução da acuidade auditiva periférica e que há uma grande incidência e correlação das questões emocionais com a percepção e/ou catastrofização do sintoma^(24)^. As taxas metabólicas, endócrinas, nutricionais e dislipidemias alteradas também são relatadas^(25)^. Aproximadamente cerca de 43% dos indivíduos com zumbido não pulsátil são diagnosticados com zumbido do tipo somatossensorial^(26)^. Deste modo, os achados corroboram com a incidência encontrada na aplicação do PIC-z, demonstrando que este é capaz de mensurar os agentes etiológicos.

Em relação às características audiológicas, ocorreu uma maior incidência de perdas auditivas sensorioneurais de grau leve ou nas frequências agudas, observadas na Tabela 4. Tais achados, já foram evidenciados por outros autores, os quais são justificados pelas características da amostra (idade dos participantes e percepção do sintoma)^(1)^. Ainda, pelo local de coleta do estudo ser em um centro de diagnóstico audiológico, dos quais os pacientes que buscam atendimento já apresentam queixas otológicas.

Embora o zumbido seja principalmente um sintoma de um problema auditivo, como perda auditiva ou exposição a ruídos em elevados níveis de pressão sonora, fatores emocionais e psicológicos podem ter um impacto significativo na percepção e na intensidade do zumbido^(21)^. Destaca-se na presente pesquisa a alta incidência de fatores ansiosos e depressivos, normalmente associados, observados nas Tabelas 5 e 6. Essas alterações, são justificados pelas mudanças nas vias neurais e hormonais, que alteram o funcionamento dos eixos corporais, ocasionando mudanças no funcionamento cerebral e a ativação de sistemas responsáveis pela regulação do humor, processo atencional e de responsividade neural, correlacionando-se a percepção e catastrofização do sintoma. A alta incidência, ainda, pode ser justificada pelo período pós pandêmico, que, por meio dos fatores de incerteza, medo e estressantes, questões emocionais tornaram-se mais evidentes^(27)^.

Estudos demonstram que alterações laboratoriais em pacientes com zumbido podem variar dependendo do fator etiológico subjacente e das comorbidades associadas. Algumas pesquisas evidenciaram que pode haver correlações com certas condições médicas que afetam os resultados laboratoriais, relacionadas à glicose, hormônio tireoidiano, vitaminas, minerais e/ou ao colesterol e triglicerídeos^(4,28)^. Na presente pesquisa, na Tabela 3, observou-se que mais de 90% da amostra, dentre os sujeitos que realizaram a avaliação, apresentaram alterações laboratoriais. Dessas, com maiores prevalências de alterações relacionadas ao colesterol, hemograma e vitamina D (Tabela 7). Assim, a avaliação sugerida demonstra a importância da mensuração laboratorial, possibilitando a orientação e encaminhamento para o manejo clínico por profissional especializado. Destaca-se, ainda, que minimiza encaminhamentos desnecessários, evitando insatisfação do sujeito e prolongamento do processo de intervenção.

Na avaliação somatossensorial, foi observada nas Tabelas 8 e 9 uma menor incidência dos participantes para este subgrupo, pois ao correlacionar a anamnese com a avaliação, permite ao clínico uma precisão, sensibilidade e uma especificidade no diagnóstico^(11)^. No estudo foram evidenciadas maiores queixas referentes a dor no pescoço/mandíbula e sintomas de disfunção temporomandibular^(29)^, com incidência de modulação nos músculos temporal, masseter, tendão da ATM, mastóide, suboccipitais e trapézio. Ainda, modulações semelhantes nos movimentos mandibulares, cervicais e oculares. Portanto, salienta-se que esses resultados demonstram que, ao associar a anamnese de investigação com as avaliações, é de suma importância para diagnóstico assertivo e para efetividade do tratamento da fisioterapia cervicocraniomandibular.

A literatura especializada apresenta uma forte recomendação de que o diagnóstico de zumbido vascular seja realizado por meio da investigação da percepção do sintoma e pelos sinais e sintomas clínicos vasculares e cardíacos associados, confirmados por meio de exames de imagem^(13)^. Normalmente, os pacientes pertencentes a este subgrupo apresentam um zumbido específico, de manifestação pulsátil, grave e ritmo com a frequência cardíaca ou mudança da pressão arterial^(13)^. Deste modo, indivíduos com zumbido vascular já estão em acompanhamento ou procuram o médico para diagnóstico, recebendo a intervenção inicial antes de buscar atendimento com outros profissionais, justificando a sua ausência no presente estudo.

Na avaliação vascular, não foram evidenciados participantes pertencentes a este subgrupo. Dos participantes, na anamnese, não foram demonstrados sinais cardíacos e/ou vasculares graves, apenas Hipertensão Arterial Sistêmica (HAS), que estava controlada no momento. Um indivíduo dos avaliados obteve modulação nas quatro avaliações. No entanto, o mesmo estava no subgrupo somatossensorial, devido a presença de zumbido pulsátil de origem somatossensorial por mioclonia. Salienta-se assim a importância de manter as avaliações, tendo em vista que ao excluir tais fatores vasculares contribuí para o diagnóstico diferencial.

Portanto, o PICz foi criado e validado. Com ele, torna-se possível a aplicação de um documento único de avaliação do zumbido, que possibilita ao profissional, tanto clinicamente, quanto cientificamente, mensurar todos os subgrupos de forma efetiva. A separação dos grupos auxilia no direcionamento da investigação, reabilitação e tratamento, porém, estes conceitos devem ser bem estabelecidos devido à incidência de associação de geradores e amplificadores do sintoma. Neste sentido, a realização do diagnóstico e encaminhamentos para tratamento dos fatores etiológicos, preferencialmente, devem ser realizados por uma equipe multidisciplinar^(4)^. Deste modo, compreendendo essa sobreposição e superada a dificuldade do diagnóstico dos fatores envolvidos na origem do zumbido, a próxima etapa consiste em uma arte: o tratamento^(30).^

### Limitações do estudo e perspectivas futuras

Destaca-se como limitações do estudo o pequeno n amostral na realização da avaliação metabólica. Sugere-se como possibilidades de estudos futuros ou em seu uso clínico a adaptação para o uso digital do protocolo, por meio de formulários eletrônicos, como uma possibilidade para ampliar seu alcance e facilitar sua implementação na prática clínica. Salienta-se, ainda, que apesar de uma casuística representativa, há a necessidade de maiores estudos, aumentando a casuística, bem como a aplicação em diferentes populações, por exemplo, no público infantil e adolescente.

## CONCLUSÃO

O Protocolo de Investigação Clínica do Zumbido foi finalizado e seu conteúdo validado, sendo possível aplicar em indivíduos adultos e idosos com percepção do zumbido. Destaca-se sua maior relevância e aplicabilidade no transtorno do zumbido.
